# The Chemokine CCL5 Inhibits the Replication of Influenza A Virus Through SAMHD1 Modulation

**DOI:** 10.3389/fcimb.2021.549020

**Published:** 2021-08-13

**Authors:** Thauane Silva, Jairo R. Temerozo, Gabriele do Vale, André C. Ferreira, Vinícius Cardoso Soares, Suelen Silva Gomes Dias, Gabriela Sardella, Dumith Chequer Bou-Habib, Marilda Siqueira, Thiago Moreno L. Souza, Milene Miranda

**Affiliations:** ^1^Laboratory of Respiratory Viruses and Measles, Oswaldo Cruz Institute (IOC), Fiocruz, Rio de Janeiro, Brazil; ^2^Laboratory on Thymus Research, Oswaldo Cruz Institute (IOC), Fiocruz, Rio de Janeiro, Brazil; ^3^National Institute for Science and Technology on Neuroimmunomodulation, Oswaldo Cruz Institute (IOC), Fiocruz, Rio de Janeiro, Brazil; ^4^Iguaçu University, Nova Iguaçu, Brazil; ^5^Laboratory of Immunopharmacology, Oswaldo Cruz Institute (IOC), Fiocruz, Rio de Janeiro, Brazil; ^6^Center for Technological Development in Health (CDTS), Fiocruz, Rio de Janeiro, Brazil; ^7^National Institute for Science and Technology on Innovation on Diseases of Neglected Populations (INCT/IDPN), Center for Technological Development in Health (CDTS), Fiocruz, Rio de Janeiro, Brazil; ^8^Program of Immunology and Inflammation, Federal University of Rio de Janeiro, UFRJ, Rio de Janeiro, Brazil; ^9^Laboratory of Neurochemistry, Biophysics Institute, Federal University of Rio de Janeiro (UFRJ), Rio de Janeiro, Brazil

**Keywords:** influenza, restriction factors, SAMHD1, CCL5/RANTES, CCR5

## Abstract

Influenza A virus (IAV) is the main etiological agent of acute respiratory tract infections. During IAV infection, interferon triggers the overexpression of restriction factors (RFs), the intracellular antiviral branch of the innate immune system. Conversely, severe influenza is associated with an unbalanced pro-inflammatory cytokine release. It is unclear whether other cytokines and chemokines released during IAV infection modulate RFs to control virus replication. Among the molecules enhanced in the infected respiratory tract, ligands of the CCR5 receptor play a key role, as they stimulate the migration of inflammatory cells to the alveoli. We investigated here whether ligands of the CCR5 receptor could enhance RFs to levels able to inhibit IAV replication. For this purpose, the human alveolar basal epithelial cell line (A549) was treated with endogenous (CCL3, CCL4 and CCL5) or exogenous (HIV-1 gp120) ligands prior to IAV infection. The three CC-chemokines tested reduced infectious titers between 30% to 45% upon 24 hours of infection. Eploying RT-PCR, a panel of RF mRNA levels from cells treated with CCR5 agonists was evaluated, which showed that the SAMHD1 expression was up-regulated four times over control upon exposure to CCL3, CCL4 and CCL5. We also found that IAV inhibition by CCL5 was dependent on PKC and that SAMHD1 protein levels were also increased after treatment with CCL5. In functional assays, we observed that the knockdown of SAMHD1 resulted in enhanced IAV replication in A549 cells and abolished both CCL5-mediated inhibition of IAV replication and CCL5-mediated cell death inhibition. Our data show that stimuli unrelated to interferon may trigger the upregulation of SAMHD1 and that this RF may directly interfere with IAV replication in alveolar epithelial cells.

## Introduction

Severe acute respiratory tract infections (SARI) represent the fourth most common cause of death in the world (World Health Organization, 2020). This public health problem leads to 4.5 million deaths per year and morbidity, leading to a substantial public health burden. Respiratory viruses are the main etiological agents of SARI (Pichon, 2017). The influenza A virus (IAV) is the most prevalent agent among these viruses, causing up to 650 thousand deaths each season (World Health Organization, 2017).

IAV is an octasegmented negative-sense single-stranded RNA virus, which infects epithelial cells in the upper respiratory tract *via* the interaction between viral hemagglutinin (HA) with the host’s sialic acid. The severe IAV infection is characterized by lysis of the alveolar cells in the lower respiratory tract, but biomarkers to predict this phenomenon may vary among studies. Nevertheless, influenza-related hospitalization and lethality are consistently associated with an uncontrolled pro-inflammatory response. During IAV-induced-cytokine storm, the protective antiviral effects promoted by interferon (IFN) are overwhelmed by other cytokines, like TNF-α and IL-6. In parallel, at the infection site, the expression of adhesion molecules is increased and chemotactic factors, such as CCL3, CCL4 and CCL5, are released, attracting leukocytes and monocytes into the alveolar lumen (Wang et al., 2012). The β-chemokines CCL3, CCL4 and CCL5 bind to the CCR5 receptor and participate in leukocyte activation, chemotaxis, cytokine secretion and cell proliferation (Velasco-Velázquez and Pestell, 2013; Flanagan, 2014). Once exacerbated, these signs may cause massive tissue damage, reducing the O2 exchange and causing respiratory distress.

Several groups of patients are considered at higher risk of influenza, like immunocompromised patients. Conversely, during the 2009 influenza pandemics, less severe events associated to Influenza A(H1N1)pdm09 were identified in HIV-1-infected individuals (Sheth et al., 2011). Indeed, we reported that the IAV replication cycle is impaired in macrophages exposed to HIV-1 or its glycoprotein gp120 *via* the IFN-induced transmembrane protein 3 (IFITM3), which acts as a cellular antiviral restriction factor (RF) (Mesquita et al., 2014). Importantly, HIV-1 gp120 is also a ligand for the CCR5 receptor (Alkhatib et al., 1996). However, it is unclear whether CCR5 activation protects or damages IAV-infected cells.

A consistent observation is the association of pleiotropic immune signals to engage the RFs. RFs are IFN-induced genes and represent an intracellular arm of the innate immune system to limit the growth of intracellular infectious agents, like viruses (Duggal and Emerman, 2012). IAV entry, replication and egress are impaired by RFs, such as, for example, IFITM, MX1 and BST-2, respectively (Xiao et al., 2013; Desai et al., 2014). Among the RFs, it is also unclear the context in which SAM domain and HD domain-containing protein 1 (SAMHD1) participates during the IAV life cycle. In this line, Sadewasser et al. (2017) did an unbiased investigation of the proteins modulated during permissive and non-permissive infections with seasonal and potentially pandemic avian IAV (Sadewasser et al., 2017). They found that SAMHD1 was downregulated during efficient propagation of seasonal IAV, whereas this RF remained unaltered throughout non-permissive infections (Sadewasser et al., 2017). Overall, these information warrant more studies on whether SAMHD1 acts as an RF against IAV.

Here, we evaluated whether CCR5 agonists induce RFs as a mechanism involved in inhibiting IAV replication in permissive cells. We found that HIV-1 gp120 and β-chemokines, especially CCL5 (also known as RANTES), enhanced SAMHD1 levels, which were consistently associated with inhibition of IAV replication. The improved comprehension of these innate signals during severe influenza may help the identification of protective biomarkers and novel antiviral targets.

## Materials and Methods

### Cells, Virus, and Critical Reagents

Human pneumocyte lineage (A549) and Madin-Darby Canine Kidney (MDCK) cells (London line) were cultured with Dulbecco’s Modified Eagle’s Medium (DMEM, Gibco), supplemented with 10% fetal bovine serum (FBS, Gibco), 100 U/mL penicillin, and 100 mg/mL streptomycin (Sigma-Aldrich, St. Louis, MO, USA). Cells were cultured at 37 °C in a 5% CO2 atmosphere. MDCK cells were kindly donated by the Centers for Disease Control and Prevention (CDC, USA), Influenza Reagent Resources (IRR) (FR-58), and used for virus propagation and titration. A549 cells were used for viral replication assays. Experiments were performed with influenza A/Switzerland/9715293/2013 (H3N2) strain, kindly provided by the CDC/IRR. IAV was grown and titrated according to the World Health Organization (WHO) manual for the laboratory diagnosis and virological surveillance of influenza (WHO, 2011). The endogenous agonists of CCR5, the β-chemokines CCL3, CCL4 and CCL5, were purchased from R&D Systems (USA), the PKC inhibitor Go6383 was purchased from Tocris (USA), and the exogenous ligand, the baculovirus-produced HIV-1 gp120 (from the CCR5-tropic HIV-1 isolate BaL) was donated from the NIH AIDS Reagent Program (NIAID/NIH, Bethesda, MD, USA). Human interferon-α (IFN-α), purchased from Sigma-Aldrich (USA), and Oseltamivir carboxylate (OST), kindly donated by Hoffman-La Roche Inc. (Basel, Switzerland), were used as positive controls. The dose of each agonist was selected based on the association constant (Kd) to activate CCR5 (Mueller et al., 2002). For CCL5, a dose-response curve was used to choose the best concentration for the influenza A inhibition effect. All these recombinant proteins were free of lipopolysaccharide (LPS), as measured by LAL-based assay (Lonza, Switzerland).

### Titration and Inhibition of Viral Replication Assay

Virus titration was performed by 50% Tissue Culture Infectious Dose (TCID50) assay with the statistical method of Reed and Muench or by real-time qRT-PCR (copies of viral RNA/mL). All reagents for real-time qRT-PCR, including primers, probes and enzymes, were used as recommended by WHO (Szretter et al., 2006). Virus quantification was based on a standard curve method (Blachere et al., 2007). A549 cells (3 × 104) were seeded into 24-well culture plates (flat bottom) and grown for 24 h at 37°C in 5% CO2. Cells were infected with three different MOIs, the cell culture supernatants were harvested after 24 hours, and virus growth was analyzed by TCID50 assay. Next, A549 cells were exposed to CCL3 (30 ng/mL), CCL4 (15 ng/mL), CCL5 (7.5, 15 or 30 ng/mL), HIV-1 gp120 (5 μg/mL), human interferon-α (IFN-α) (10 ng/mL) or OST (0.5 µg/mL) for 24 h, and then infected at MOI of 2 for 1 h at 37°C and 5% CO2. Non-internalized viruses were removed by washing, and cell monolayers were replenished with fresh medium with 1.5 μg/mL TPCK-trypsin. At 24 h post-infection (hpi), the supernatants were harvested to quantify influenza titers by TCID50 assay.

### Quantitative Analysis of RF mRNA Levels

A549 cells (3.0 × 10^5^) were seeded into 24-well culture plates (flat bottom) and maintained overnight at 37°C in 5% CO_2_. Then, they were exposed to CCL3 (30 ng/mL), CCL4 (15 ng/mL), CCL5 (15 ng/mL), HIV-1 gp120 or IFN-α (10 ng/mL) for 24 h. Cells were lysed and total RNA was extracted using a commercial kit (RNeasy mini kit; Qiagen). cDNA was synthesized with RT2 First Strand kit (Qiagen, USA). The mRNA levels for the RFs were evaluated by the RT2 Profiler PCR Array (CAPH12347A) (Qiagen, USA), composed of primers for different IFN-induced restriction factors, such as Tetherin (cat # PPH05790B), IFITM1 (cat # PPH05981C), IFITM2 (cat # PPH05548F), IFITM3 (cat # PPH02872E), MX1 (cat # PPH01325A), MX2 (cat # PPH01326F), SAMHD1 (cat # PPH18140A), APOBEC3G (cat # PPH06904A), MCPIP1 (cat # PPH16134B), and IFN1-α (PPH01321B), IFN receptor (PPH00869F) and the housekeeping gene GAPDH. Results were normalized to GAPDH gene mRNA levels. The transcriptional levels were analyzed by calculation 2 (-ΔΔCT), where ΔΔCT = ΔCT (experimental) - ΔCT (control).

### Immunoblot Assays

To quantify the protein levels of SAMHD1, cells were lysed using RIPA buffer containing protease and phosphatase inhibitors (Sigma-Aldrich, USA). A total of 50 µg of the extracted proteins (Qubit Protein assay kit, Invitrogen) was prepared with Laemmli’s buffer and separated by 12% sodium dodecyl sulfate (SDS)–polyacrylamide gel electrophoresis and transferred to a nitrocellulose membrane, which was blocked with BSA 5% for 1 h. Specific proteins were detected using anti-SAMHD1 (Thermofisher, USA, #MA5-25354 or #MA5-25298), Phospho-SAMHD1 (Cell Signaling Technology, USA, #89930) or mouse polyclonal anti-β-actin antibody (Sigma-Aldrich, USA, #A1978) during overnight incubation, following incubation with secondary antibody labeled with horseradish peroxidase IRDye 800CW Goat Anti-Mouse IgG (LI-COR). All antibodies were diluted in blocking buffer. The detections were performed by Supersignal Chemiluminescence (GE Healthcare, USA) or by fluorescence imaging using the Odyssey system, and densitometry analysis was performed using ImageJ software (Version 1.6.0). Densitometry values were initially expressed as arbitrary units to calculate mean values. Next, densitometry values for the control bands (medium only) were normalized to be 1.0. Therefore, the values for the other bands were expressed as n-fold change relative to control bands.

### Liposomal Efficiency Delivery Assay and SAMHD1 Knockdown

A549 cells were seeded into glass coverslips in 24-well culture plates at 3 × 104 (for immunofluorescence analysis) or seeded into 6-well culture plates at 5 x 105 (for western blot analysis) and grown for 24 h at 37°C in 5% CO2, without antibiotics. Cells were transfected for 6 hours with BLOCK-iT™ Alexa Fluor™ Red Fluorescent Control (Thermo Fisher, USA #4750100) at 100 nM (for liposomal efficiency delivery assays) or siRNA targeting SAMHD1 or its scramble sequence (Thermo Fisher, USA, Silencer^®^ Select #24790) at different concentrations (for SAMHD1 knockdown assays) in Opti-MEM (Gibco, USA), using Lipofectamine 2000 (Sigma-Aldrich, USA). For liposomal efficiency delivery assays, after the transfection incubation period, cells were washed and maintained in culture for 18 hours and then processed for immunofluorescence analysis. The efficiency delivery was obtained by (AF555 positive cell number divided by total cell number) x 100 in 4 different fields. In SAMHD1 knockdown assays, after the transfection incubation period, cells were washed, treated or not with CCL5 or IFN-α for 18 hours and then processed for immunofluorescence or western blot analysis. For evaluation of SAMHD1 participation in CCL5-mediated IAV inhibition, SAMHD1-silenced cells were treated or not with CCL5 or IFN-α for 18 hours and then infected with IAV at MOI 2 for 1 hour, at 24 hpi supernatants were harvested to quantify IAV replication by real-time qRT-PCR.

### Immunofluorescence Analysis

Cells were washed with PBS and fixed with 2% paraformaldehyde for 15 min and permeabilized with 0.3% Triton-X 100 for 5 min, following overnight incubation with mouse anti-SAMHD1 primary antibody (Thermofisher, #MA525354) at 4°C. Later, cells were rinsed three times and exposed to Alexa Fluor 488 Goat Anti-Mouse IgG (H+L) (Thermofisher, #A32723) for 2 h at room temperature, following by washing. Cell nuclei were stained with DAPI (4’,6-diamidino-2-phenylindole) for 5 min and the coverslips were mounted in n-propyl gallate. Staining was also performed without primary antibodies to monitor background signal. Images were obtained with a 40X objective in an Olympus BX60 fluorescent microscope (Japan). The relative fluorescence intensity (RFI) was calculated by the average ratio between the total intensity over the total number of nuclei in 4 different fields with the same increase.

### LDH Measurement

Cell death was determined according to the activity of lactate dehydrogenase (LDH) in the culture supernatants using a CytoTox^®^ Kit (Promega, USA) according to the manufacturer’s instructions. Results are expressed as OD at 490 nm obtained from reads in a SpectraMax M2 microplate reader (Molecular Devices, USA).

### Statistical Analysis

The data were analyzed using GraphPad Prism 8.0 software, and the determination of the significance between the different experimental groups was performed by one-way ANOVA with Tukey post-test. Results are presented as the mean ± SD with a confidence interval of 95%, and significant p values were represented as * for <0.05, ** for <0.01 and *** for <0.001.

## Results

### CCR5 Ligands Reduce IAV Replication and Enhance SAMHD-1 Levels

**Figure 1:**
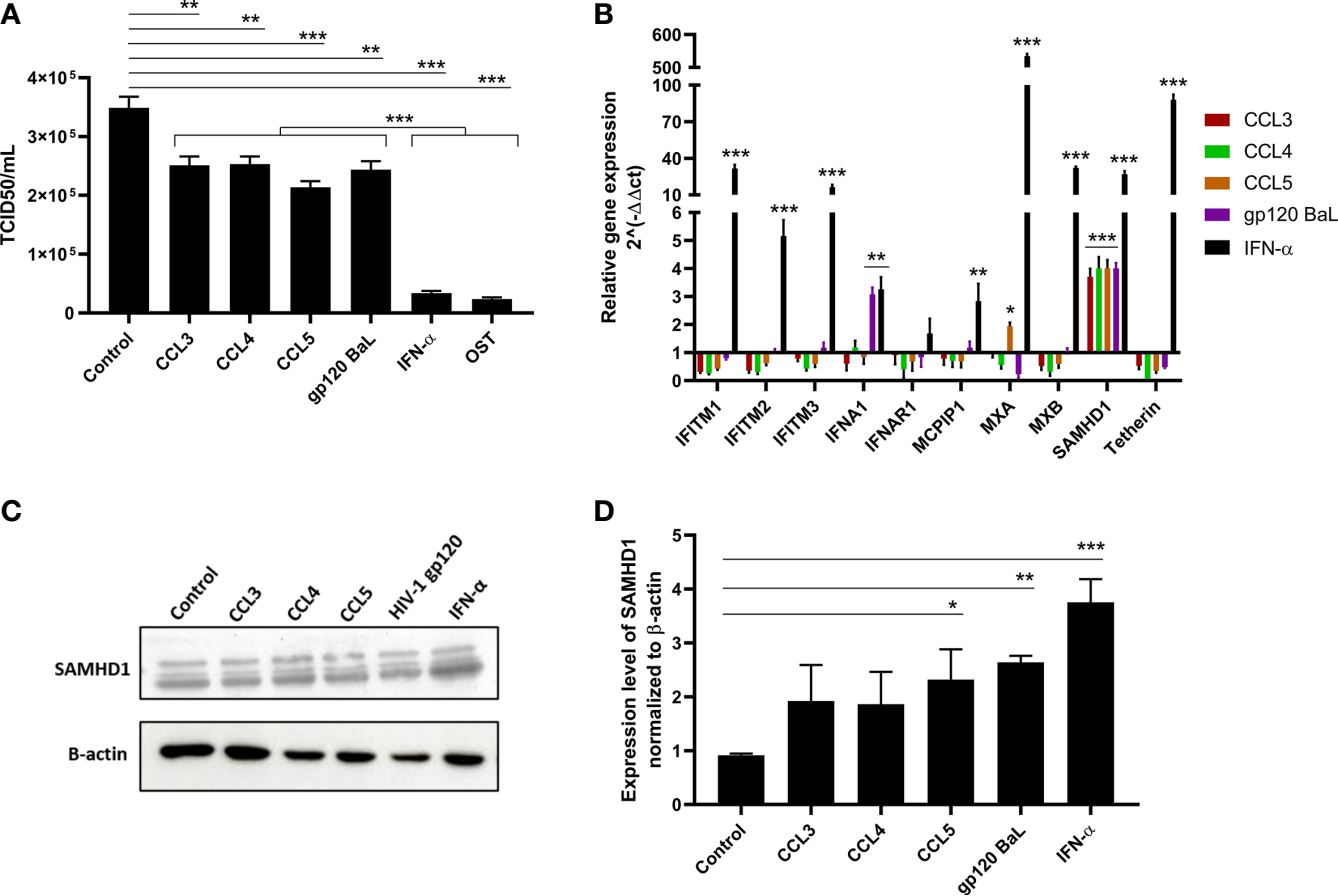
CCR5 receptor agonists inhibit influenza A replication and enhance transcriptional and protein levels of SAMHD1. **(A)** A549 cells were exposed to CCL3 (30 ng/mL), CCL4 (15 ng/mL), CCL5 (15 ng/mL) or HIV-1 gp120 Bal (5 µg/mL); IFN-α (10 ng/mL) and OST (0.5 µg/mL) were used as positive control. After 24 hours, cells were infected with influenza A virus at MOI 2 for 1h, and the supernatant was harvested at 24 hpi. The viral titer was evaluated by TCID_50_ assays. **(B)** For transcriptional analysis, total RNA of A549 cells exposed to agonists for 24 hours was extracted and the relative expression of restriction factors was determined using customized RT² Profiler PCR Array. Analysis for relative gene expression was performed using the 2^(-ΔΔCT) method, with GAPDH as reference gene. **(C, D)** Protein expression of SAMHD1 was evaluated by western blot assays using β-actin as a housekeeping protein; bands were analyzed by densitometry using the ImageJ software 1.5.0, with **(D)** representing three blot assays. The basal levels of SAMHD1 protein are shown in A549 cells (control), exposed to medium only. Data were obtained from 3 independent experiments with technical duplicates. *p < 0.05, **p < 0.01 and ***p < 0.001.

First, we aimed to determine the best MOI for IAV infection of A549 pneumocyte cell line and found that MOIs equal to 2 and 3 were significantly different from 1, and while the titer peak was in MOI 3, the range of replication was similar (**Supplementary Figure 1**). A549 cells were treated with endogenous (CCL3, CCL4 and CCL5) and exogenous (gp120 HIV-1 BaL) CCR5 ligands and then infected with IAV at an MOI of 2. We observed that all CCR5 agonists inhibited viral replication between 30% to 45% (**Figure 1A**) and, as expected, the positive controls IFN-α and OST potently reduced IAV replication. We next evaluated whether those ligands could increase the mRNA levels of RFs, as a possible mechanism involved with the inhibitory effect triggered by β-chemokines towards IAV replication. Among the evaluated RFs, only SAMHD1 was induced upon β-chemokine stimuli, showing a 4-fold enhancement of mRNA levels, compared to untreated cells (**Figure 1B**). As a positive control, IFN-α induced all studied transcripts (**Figure 1B**). To confirm these results at protein levels, we performed immunoblotting on A549 cells exposed to CCR5 ligands. Western blotting analysis showed that the β-chemokines induced a 2-fold and the HIV-1 gp120 a 3-fold increase in SAMHD1 levels upon cell treatment, compared to control (medium only) (**Figures 1C, D**). IFN-α augmented by 4-times the expression of SAMHD1, compared to the control cells.

**Figure 2:**
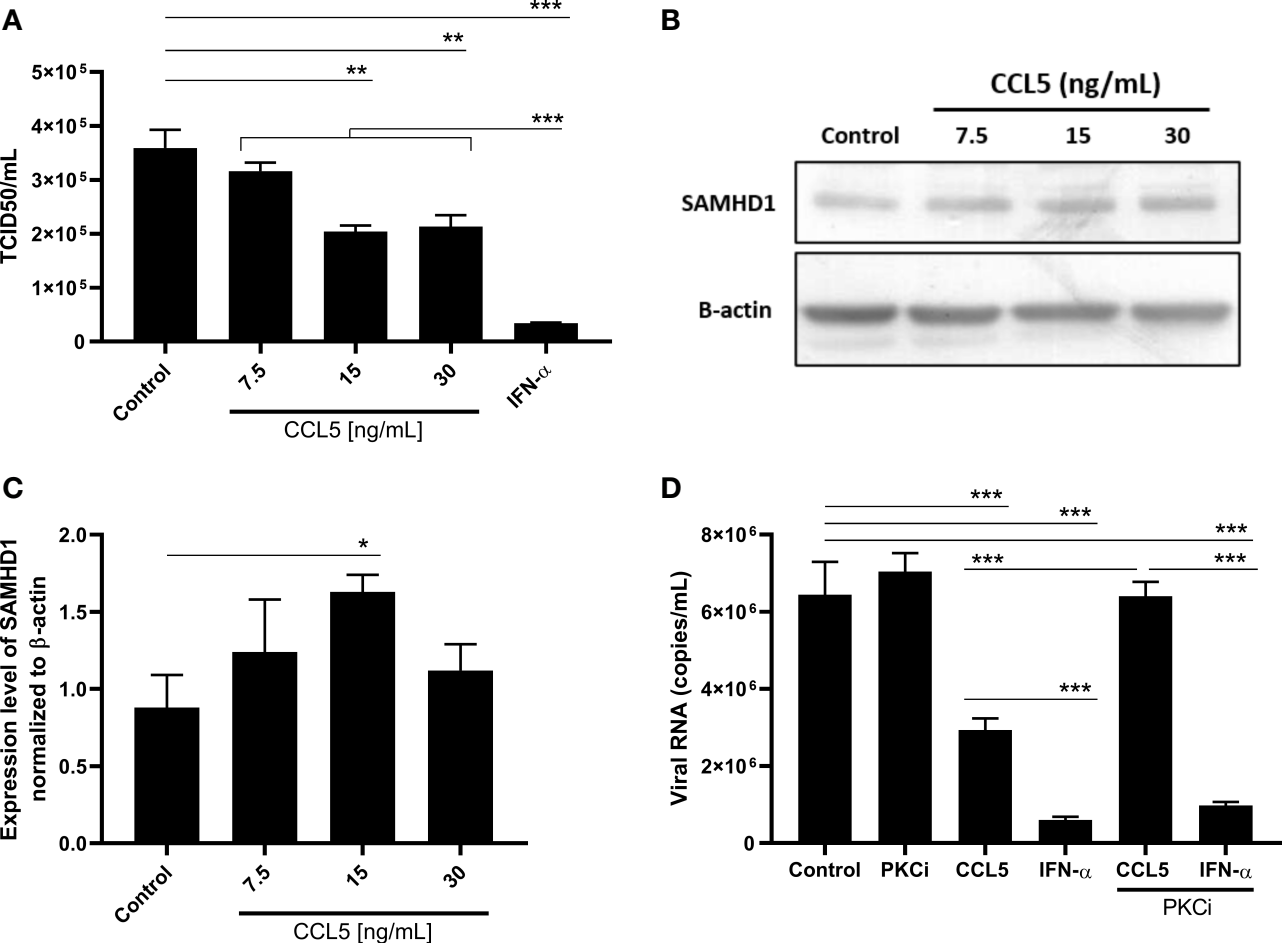
CCL5 inhibits influenza A replication and induces SAMHD1 protein expression. A549 cells were treated with different concentrations of CCL5 and, 24 hours later, were **(A)** infected with influenza A at MOI 2 during 1h for viral replication analysis, or **(B, C)** lysed for SAMHD1 protein expression evaluation by western blot assay with β-actin as a housekeeping protein; bands were analyzed by densitometry using the ImageJ software 1.5.0, with **(C)** representing three blot assays. The basal levels of SAMHD1 protein are shown in A549 cells (control) exposed to medium only. **(D)** A549 cells were treated with CCL5 (15 ng/mL) in the presence or not of a PKC inhibitor (Go6983, 50 nM), and 24 hour later were infected with influenza A at MOI 2 during 1 hour, for viral replication analysis. Viral titer was evaluated 24 hpi by TCID_50_ assays. Data were obtained from 3 independent experiments with technical duplicates. *p < 0.05, **p < 0.01 and ***p < 0.001.

Because CCL5 showed, in conjunction, the best results for influenza inhibition and SAMHD1 enhancement levels, we selected this β-chemokine to continue the study. A dose-response curve confirmed that 15 ng/mL of CCL5 was optimal to inhibit IAV replication, achieving 45% of viral inhibition (**Figure 2A**), and to induce SAMHD1 (**Figures 2B, C**). This concentration is also the optimal dose to activate CCR5 (Mueller et al., 2002). Given that PKC is one of the major transducers of CCR5 signaling (Oppermann, 2004), we confirmed that the engagement of this receptor and the signal transduction triggered the inhibition of IAV replication by β-chemokines, since the phenomenon was abrogated in the presence of a PKC inhibitor, while no changes were observed in IFN-α-treated cells (**Figure 2D**).

### Downmodulation of SAMHD1 Enhances IAV Replication

**Figure 3:**
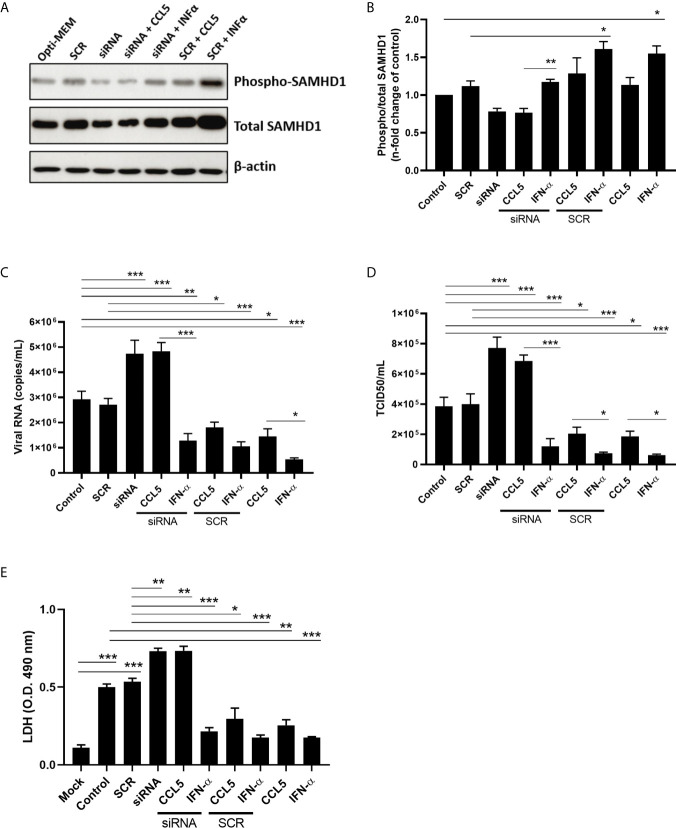
SAMHD1 knockdown elicits influenza A replication and abolishes CCL5 inhibitory effect on viral replication. A549 cells were transfected with siRNA for SAMHD1 or its scramble sequence at a concentration of 100 nM in Opti-MEM, using Lipofectamine 2000. At 6 hours post transfection, A549 cells were exposed or not to CCL5 (15 ng/mL) or IFN-α (10 ng/mL). After 18 h of exposure **(A, B)**, monolayers were lysed with RIPA buffer for western blot assay to SAMHD1 and β-actin, as a housekeeping protein, or **(C, D)** infected with influenza A virus at MOI 2. At 24 hpi, supernatants were harvested for extraction of viral RNA and the viral titer measured through qRT-PCR (copies/mL) and by TCID_50_ assays. **(E)** Supernatants of those cultures were also quantified for LDH activity, as a marker of cell death. Data were obtained from 3 independent experiments with technical duplicates. *p < 0.05, **p < 0.01 and ***p < 0.001.

In order to assess the sensitivity of IAV to SAMHD1, we silenced the expression of *SAMHD1* gene transfecting A549 cells with a commercial specific siRNA. The liposomal delivery was evaluated by immunofluorescence, showing a mean delivery efficiency of 68% (± 7.3%) (**Supplementary Figure 2**). The efficiency of SAMHD1 knockdown was accessed by immunoblotting (western blot - WB) and immunofluorescence (IFA) assays. We verified that siRNA against SAMHD1 at 100 nM was able to reduce more than 60% of the protein content in WB (**Supplementary Figure 3A**) and up to 50% in IFA assays (**Supplementary Figures 3B, C**). Next, we verified whether CCL5 treatment, besides increasing SAMHD1 expression, also enhanced its activation (phospho/total ratio) in IAV-infected cells and whether the modulation of SAMHD1 was involved in the inhibitory mechanism of CCL5 on IAV replication. We found that only the positive control IFN-α induced the activation of SAMHD1, and that this effect was abrogated in siRNA-transfected cells (**Figures 3A, B**). However, while we observed no changes in the activation profile of SAMHD1 in IAV-infected cells exposed to CCL5 (**Figures 3A, B**), we verified that the reduced SAMHD1 protein levels by siRNA allowed an increase of IAV replication compared to scramble and control, which was not reverted in CCL5-treated cells (**Figures 3C, D**). We also found that, besides inhibiting IAV replication, CCL5 also reduced the IAV-induced cell death, and that this cellular protection was also abrogated by the knockdown of SAMHD1 (**Figure 3E**). As a control, IFN-α inhibited viral replication and cell death regardless of SAMHD1 levels (**Figures 3C–E**). Taken together, our data show that through direct participation of the viral restriction factor SAMHD1, the β-chemokine CCL5 inhibits IAV replication in alveolar epithelial cells.

## Discussion

Annually, the influenza A virus causes seasonal infections which affect people in any age group, being more severe for the elderly and others with comorbidities. Although vaccination is the most effective way to reduce the impact of influenza infections, this virus may escape the immune system. Complementary, antiviral drugs, such as oseltamivir (OST), may limit disease progression to a severe form. Nevertheless, 1-2% of influenza strains are OST-resistant (Dixit et al., 2013). Improved comprehension of the pathophysiology to identify biomarkers of disease progression/protection and novel antiviral targets are necessary. RFs are a class of antiviral proteins induced by IFN that may provide protection during influenza infection (Villalón-Letelier et al., 2017). In the upper respiratory tract, IAV-infected cells trigger the IFN type I signaling pathway leading to the transcription of RFs, among other proteins. IFITM3 is a well-known RF to block IAV entry into the host cell *in vivo* and *in vitro* (Everitt et al., 2012; Zani and Yount, 2018).

In our previous work, we observed that during HIV-1 and influenza A(H1N1)pdm09 experimental co-infection, HIV-1 particles or HIV-1 surface glycoprotein (gp120), an exogenous CCR5 agonist, reduced influenza replication in an IFITM3-dependent fashion (Mesquita et al., 2014). In agreement with these results, HIV-1-infected individuals affected by A(H1N1)pdm09 infection presented benign clinical influenza outcomes, depending on their progression to AIDS (Barchi et al., 2010; Osmsby et al., 2011). Therefore, it is plausible to hypothesize that endogenous and exogenous CCR5 agonists could inhibit influenza virus replication.

We show here that CCR5 ligands trigger an anti-IAV effect by reducing virus replication in a human-derived pneumocyte cell line. We observed that SAMHD1 is up-regulated at transcription and translational levels. Knockdown of SAMHD1 prevented the antiviral activity of CCL5, which was not observed in cells exposed to interferon. The interferon-stimulated genes (ISGs) encode multiple antiviral proteins with diverse modes of action, therefore, promote a broader activity than CCR5 induction (Shim et al., 2017). Then, the interferon inhibition efficiency is higher than that observed for CCR5 agonists.

The cell restriction factor SAMHD1 is associated with cell proliferation, immune responses and virus restriction (Ballana and Esté, 2015). However, the mechanism underlying SAMHD1 virus restriction is controversial; both dNTPase and RNase functions have been suggested as essential steps for its antiviral activity, but the exonuclease activity cannot be associated with the SAMHD1 active site (Tüngler et al., 2013; Hansen et al., 2014; Ballana and Esté, 2015). The enzyme activity is regulated by the binding of dGTP to SAMHD1 allosteric sites (Hansen et al., 2014). At least one of these activities is required for inhibition of HIV, Simian Immunodeficiency Virus, herpes simplex virus type 1, and the porcine reproductive and respiratory syndrome virus replication (Lahouassa et al., 2012; Gramberg et al., 2013; Yang et al., 2014). However, regarding arboviruses replication, up-regulation of SAMHD1 is positively associated with harmful infection (Wichit et al., 2019). The role of SAMHD1 in IAV replication requires further investigation, since IAV permissive cells, which can produce infectious virus progeny, display reduced SAMHD1 levels (Sadewasser et al., 2017). In line with that, our siRNA assays to knockdown SAMHD1 strengthened that this protein is negatively associated with IAV replication.

Taken together, our data provide experimental findings to demonstrate that the common signal during influenza infection, the up-regulation of β-chemokines, which are the preferred ligands to CCR5 to attract inflammatory cells to the site of infection, engages SAMHD1 expression, as a contributive response to reduce IAV replication. Therefore, the results presented here open perspectives for new studies on the signaling modulation through cellular receptors to induce restriction factors capable of controlling infections that burden the public health system.

## Data Availability Statement

The original contributions presented in the study are included in the article/**Supplementary Material**. Further inquiries can be directed to the corresponding author.

## Author Contributions

TS conceived, designed and performed the experiments, analyzed the data, prepared figures, and wrote or reviewed drafts of the paper. TLS and MM conceived and designed the experiments, and wrote or reviewed drafts of the paper. GV, AF, VS, SD, and GS performed the experiments. JT designed and performed the experiments, analyzed the data, and wrote or reviewed drafts of the paper. MS and DB-H conceived and designed the experiments, contributed reagents/materials/analysis tools, and reviewed drafts of the paper. All authors contributed to the article and approved the submitted version.

## Funding

This work was supported by Conselho Nacional de Desenvolvimento Científico e Tecnológico (CNPq), Fundação de Amparo à Pesquisa do Estado do Rio de Janeiro (FAPERJ) and Instituto Oswaldo Cruz - Fiocruz.

## Conflict of Interest

The authors declare that the research was conducted in the absence of any commercial or financial relationships that could be construed as a potential conflict of interest.

## Publisher’s Note

All claims expressed in this article are solely those of the authors and do not necessarily represent those of their affiliated organizations, or those of the publisher, the editors and the reviewers. Any product that may be evaluated in this article, or claim that may be made by its manufacturer, is not guaranteed or endorsed by the publisher.
