# Supplementary material for: The Chemokine CCL5 Inhibits the Replication of Influenza A Virus Through SAMHD1 Modulation
Source: Front Cell Infect Microbiol. 2021 Aug 13;11:549020. doi: 10.3389/fcimb.2021.549020 (PMC8418070; doi:10.3389/fcimb.2021.549020)
Supplement: Supplementary file 1 [file DataSheet_1.pdf]

The chemokine CCL5 inhibits the replication of Influenza A virus through SAMHD1 modulation

Thauane Silva[1,#], Jairo R. Temerozo[2,3,#], Gabriele do Vale[1], André C. Ferreira[4,5,6], Vinícius Cardoso Soares[5,7], Suelen Silva Gomes Dias[5], Gabriela Sardella[8], Dumith Chequer Bou-Habib[2,3], Marilda Siqueira[1], Thiago Moreno L. Souza[5,6], Milene Miranda[1,*]

[1]Laboratory of Respiratory Viruses and Measles, Oswaldo Cruz Institute (IOC), Fiocruz, Rio de Janeiro, RJ, Brazil

[2]Laboratory on Thymus Research, Oswaldo Cruz Institute (IOC), Fiocruz, Rio de Janeiro, RJ, Brazil

[3]National Institute for Science and Technology on Neuroimmunomodulation, Oswaldo Cruz Institute (IOC), Fiocruz, Rio de Janeiro, RJ, Brazil

[4]Iguaçu University, Nova Iguaçu, RJ, Brazil

[5]Laboratory of Immunopharmacology, Oswaldo Cruz Institute (IOC), Fiocruz, Rio de Janeiro, RJ, Brazil

[6]National Institute for Science and Technology on Innovation on Diseases of Neglected Populations (INCT/IDPN), Center for Technological Development in Health (CDTS), Fiocruz, Rio de Janeiro, RJ, Brazil

[7]Program of Immunology and Inflammation, Federal University of Rio de Janeiro, UFRJ, Rio de Janeiro, RJ, Brazil

[8]Laboratory of Neurochemistry, Biophysics Institute, Federal University of Rio de Janeiro (UFRJ), Rio de Janeiro, RJ, Brazil

# These authors contributed equally to this work.

*** Correspondence:** Milene Miranda email: mmiranda@ioc.fiocruz.br
Av. Brasil, 4365. Manguinhos - Rio de Janeiro - RJ - Brasil, CEP: 21040-360
Pav. Hélio e Peggy Pereira (HPP) Sala B104, primeiro andar
Tel: 55 21 2562-1731

Keywords: influenza, CCR5, restriction factors, SAMHD1, CCL5/RANTES

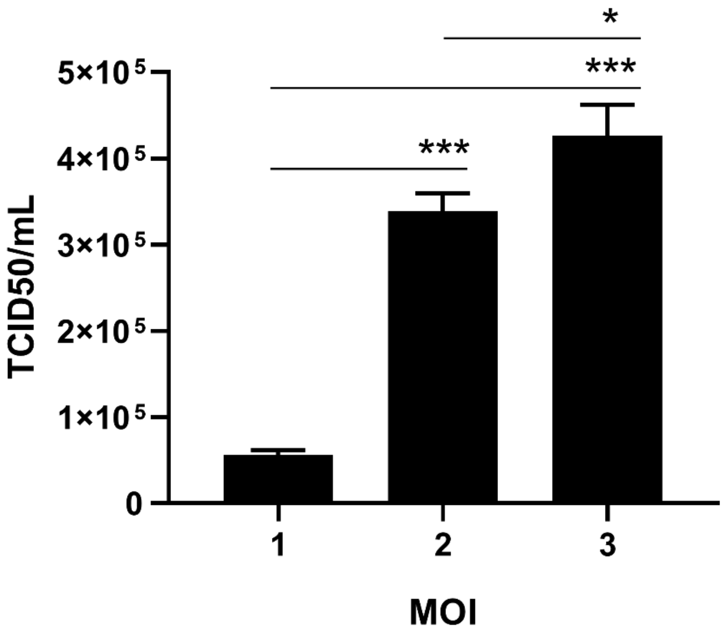

Supplemental figure 1. Evaluation of Influenza A virus yield at different MOIs. A549 cells were infected with different influenza A MOIs and virus yield was quantified at 24 hpi by TCID50 assay. Data were obtained from 3 independent experiments with technical duplicates.

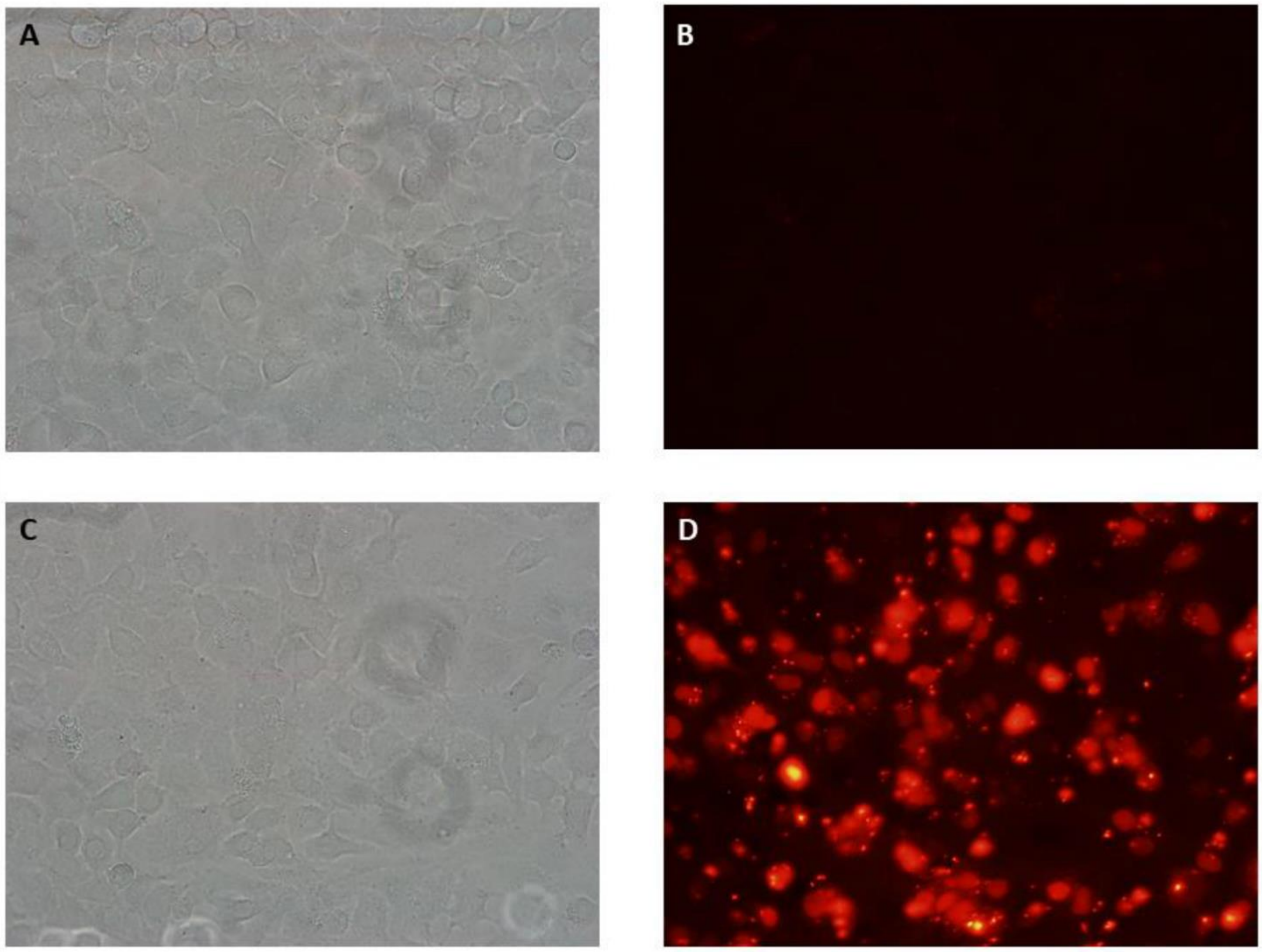

Supplemental figure 2. Evaluation of siRNA delivery efficiency. A549 cells were subjected to liposome-transfection using a fluorescent-marked siRNA nonspecific control, After 24 hours, cells were fixed with PFA 2% for 15 minutes and visualized for assessment of siRNA control delivery. **(A and C)** Bright-field images of cells exposed to Lipofectamine 2000 with **(C)** or without **(A)** siRNA control. **(B and D)** Alexa Fluor 555 fluorescent images of cells exposed to Lipofectamine 2000 with **(D)** or without **(B)** siRNA control. Images were obtained with an 40X objective in an Olympus BX60 fluorescent microscope (Japan). The delivery efficiency was obtained by (AF555 positive cell number divided by total cell number)x100 in 4 different fields. Data were obtained from one experiment with technical triplicates.

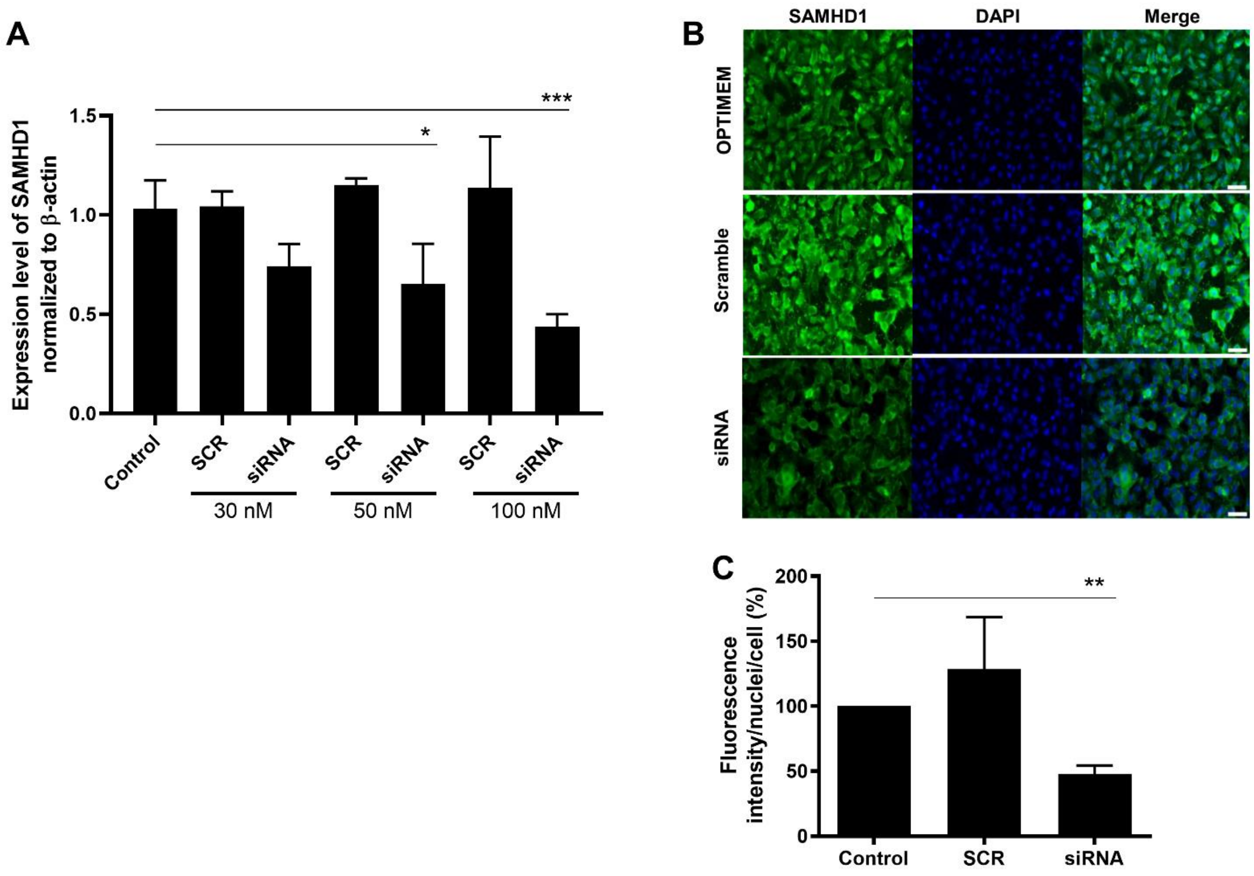

Supplemental figure 3. Knockdown of SAMHD1. A549 cells were transfected with siRNA for SAMHD1 or its scramble sequence in 30, 50 or 100 nM in Opti-MEM, using lipofectamine 2000. After 24 h, **(A)** the cells monolayers were lysed with RIPA buffer for western blot assay to phosphor (inactive) or total SAMHD1 and β-actin, as a housekeeping protein, or **(B and C)** fixed with paraformaldehyde 2% for immunofluorescence assay, as described in Materials and Methods, the relative intensity of fluorescence was calculated by the average of the ratio between the total intensity over the total number of nuclei in 4 different fields with the same increase and percentages were calculated considering Opti-MEN as 100%. Scale bars, 50 µm. Data were obtained from 4 independent experiments with technical duplicates.
